# Tracheostomy Exchange Resulting in Rare Combination of Pneumomediastinum, Pneumothorax, Massive Pneumoperitoneum, and Subcutaneous Emphysema

**DOI:** 10.7759/cureus.1489

**Published:** 2017-07-18

**Authors:** Saqib Saeed, Sara Alothman, Ali Safavi, Brian Donaldson, Alexius Ramcharan, Hector DePaz

**Affiliations:** 1 Surgery, Harlem Hospital Center

**Keywords:** tracheostomy, pneumothorax, pneumomediastinum, pneumoperitoneum

## Abstract

Tracheostomy tube change is a relatively common and simple procedure once a tract is formed between the skin and the trachea. Regular tracheostomy tube changes decrease the risk of postoperative pulmonary infection and granulation tissue formation. However, serious complications, such as loss of airway, subcutaneous emphysema, and mediastinitis, can occur if the tube exchange is performed inappropriately. We present a rare association of subcutaneous emphysema, tension pneumothorax, pneumomediastinum, and pneumoperitoneum following a tracheostomy tube exchange in a 56-year-old patient who had his tracheotomy placed a month ago. The patient was successfully managed conservatively by chest tube and supportive care.

## Introduction

A significant number of complications are associated with both open and percutaneous tracheostomy procedures [1]. Major early complications include pneumothorax, hemorrhage, and tracheal displacement, while late complications include tracheal stenosis and fistula formation. The most frequently performed procedure after tracheostomy is the tube exchange, which can be associated with establishing false tract if done prior to stomal maturity. False tract formation after inappropriate tube exchange can result in serious complications such as loss of airway, subcutaneous emphysema, mediastinitis and even death. The combination of subcutaneous emphysema, tension pneumothorax, pneumomediastinum and pneumoperitoneum after tracheostomy is rarely reported. We report a case of subcutaneous emphysema, tension pneumothorax, pneumomediastinum, and pneumoperitoneum after a tracheostomy exchange procedure.

## Case presentation

**Figure 1:**
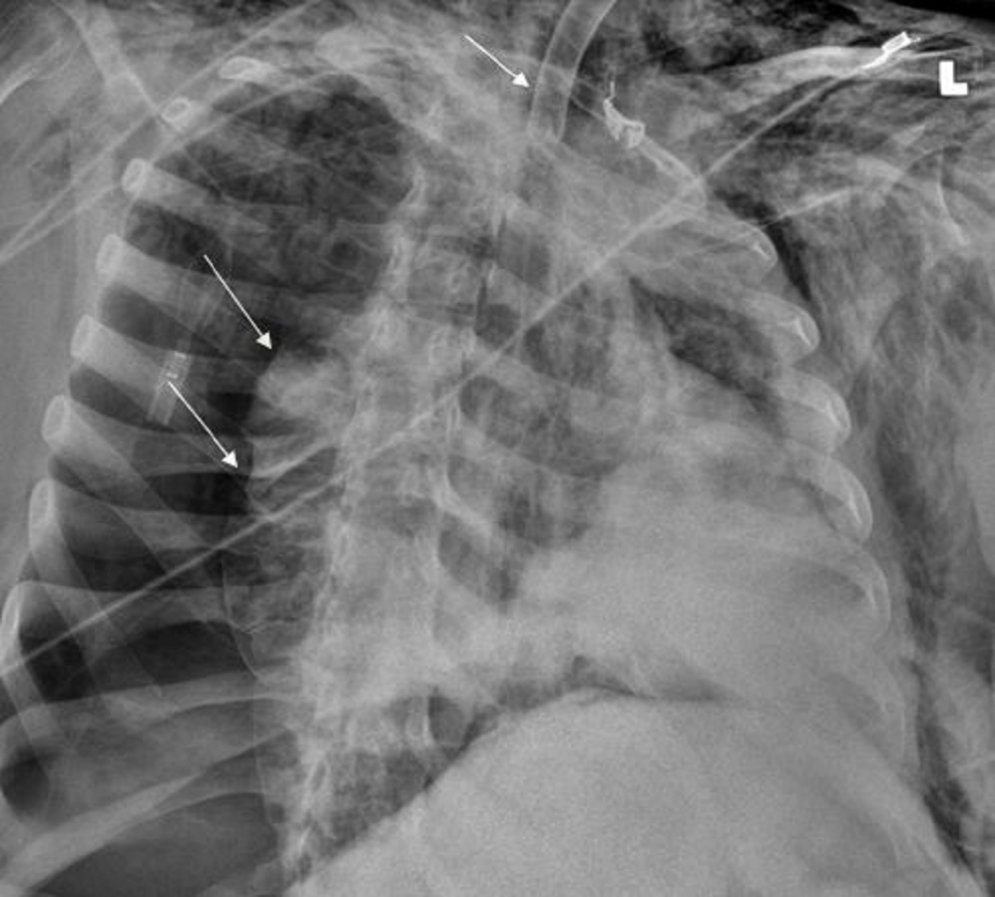
Right-sided pneumothorax as shown by the arrows (Right) on initial chest X-ray with tracheostomy tube repositioned (Top arrow)

A 51-year-old male from a nursing home with cerebral palsy and acute-on-chronic respiratory failure presented to the emergency department (ER) with acute respiratory distress. The patient had a tracheostomy placed a month prior to presentation. While at the nursing home, the patient’s non-fenestrated tracheostomy tube was exchanged for a similar sized tube secondary to clogging by secretions. Following the tube change, the patient developed acute respiratory distress requiring Ambu bagging and transfer to the ER. On arrival to the ER, his oxygen saturation was 80% and he was tachypneic. The tracheostomy tube was immediately replaced with a new tube. Initial chest X-ray at the time of the tube repositioning and replacement showed a right-sided pneumothorax, pneumomediastinum, and free air under the right hemidiaphragm (Figure 1).

**Figure 2:**
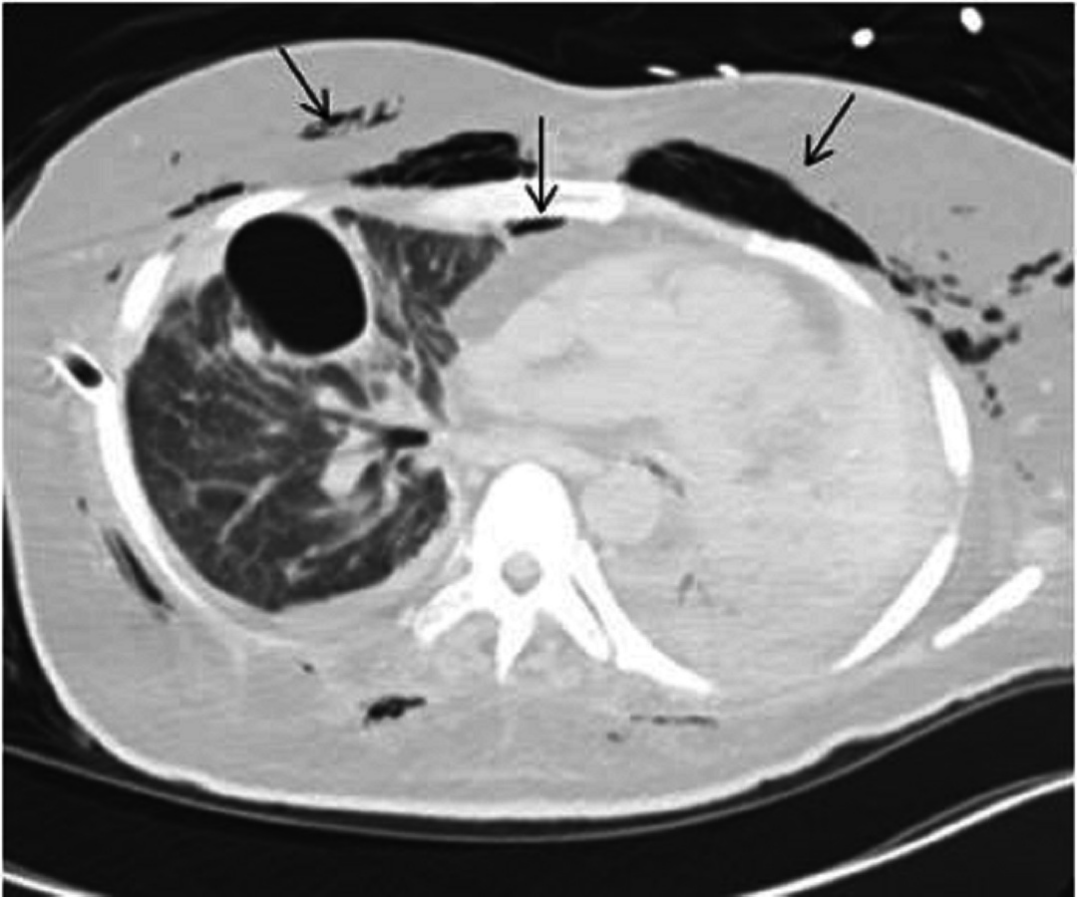
Subcutaneous emphysema and pneumomediastinum on computed tomography (CT) of the chest as indicated by arrows

**Figure 3:**
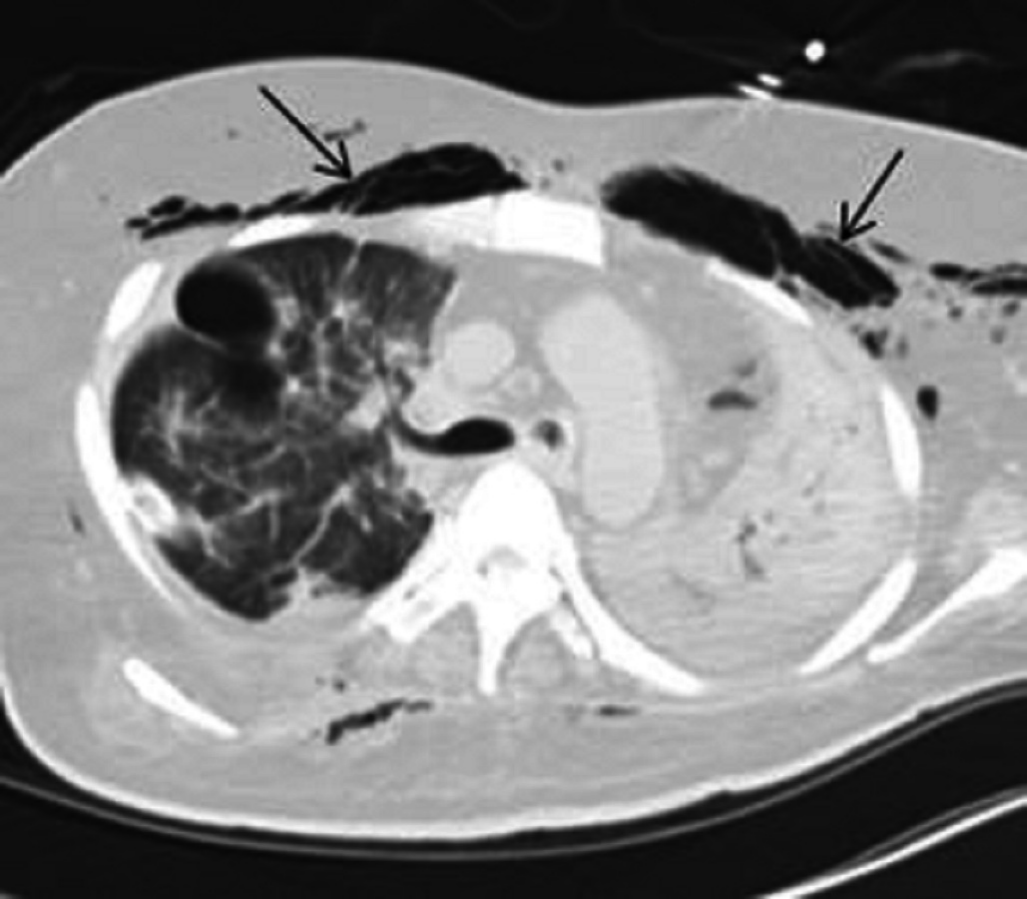
Computed tomography (CT) chest showing subcutaneous emphysema as indicated by arrows

**Figure 4:**
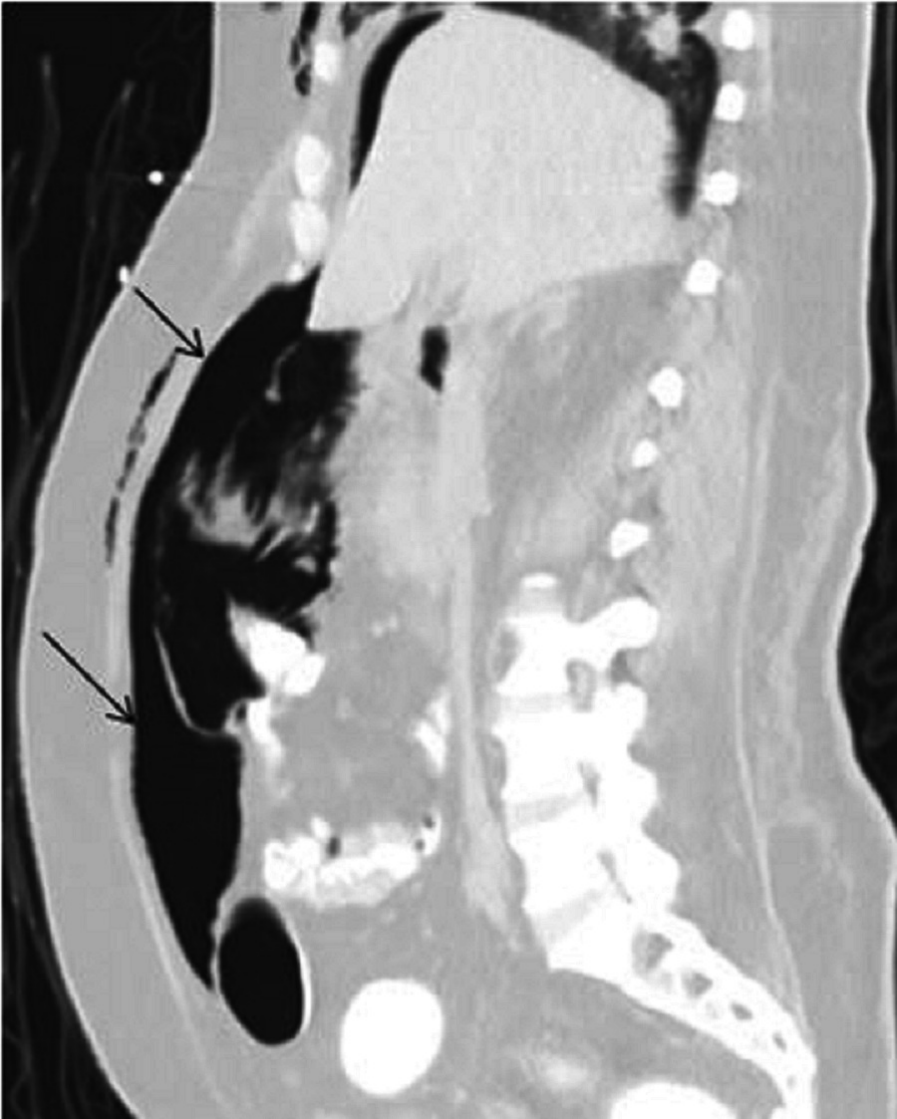
Computed tomography (CT) abdomen with a massive pneumoperitonium

A right-sided chest tube was placed in the ER. His respiratory distress resolved. The patient was placed on mechanical ventilation and transferred to the intensive care unit. Computed tomography (CT) of the chest obtained later showed subcutaneous emphysema, pneumothorax, and pneumomediastinum (Figures 2-3) while a CT of the abdomen identified a massive pneumoperitoneum (Figure 4).

Clinically, the patient remained stable. The abdominal exam didn’t show any evidence of peritonitis. The patient was managed conservatively. His condition improved remarkably. He was weaned off mechanical ventilation with normal oxygen saturation. The chest tube was removed on day 10 of hospitalization. Enteral feeding was commenced, and he was discharged back to his nursing home.

## Discussion

Several case reports have been published demonstrating the communication between the chest and abdomen through diaphragmatic apertures. Although pneumothorax, subcutaneous emphysema, pneumomediastinum, and pneumoperitoneum after tracheostomy placement have been reported, this is the first case report demonstrating these four findings in combination after the tracheotomy exchange procedure.

In the case of perforation following colonoscopy, endoscopic retrograde cholangiopancreatography (ERCP), or perforated abdominal viscus, air can track up to the mediastinum via the aortic hiatus. Once in the chest, a pneumothorax can result if high pressure ruptures the mediastinal pleura. Similarly, case reports have documented the spread of air from the chest to the abdomen following tracheal intubation and percutaneous tracheostomy.

The proposed mechanism of the spread of air from the chest to the abdomen has been the rupture of pulmonary alveoli due to extreme pressure and distention [2]. Air spreads to mediastinum by dissecting the lung hilum along the peribronchovascular sheath. Further dissection along the mediastinal fascial planes results in subcutaneous emphysema involving the cervical region and thorax. Air from the mediastinum can pass to the retroperitoneum where it can follow intestinal vessels reaching intestinal walls. The parietal pleura and transversalis fascia blends with each other at the diaphragmatic attachment at the xiphoid process and at the costal margin. This allows air to spread between the transversalis fascia and the parietal peritoneum. Also, structural weakness in the tendinous part of the diaphragm allows transdiaphragmatic migration of air between the chest and abdomen. Further high pressure can disrupt the peritoneum resulting in a pneumoperitoneum [3].

Though complications after the formation of a tracheostomy tract are extremely rare and exchange of the tracheotomy tube is a relatively simple procedure, serious manifestations can occur if handled inappropriately. Patients with unusual airway anatomy can be at greater risk of having a tube inserted, creating a false passage while performing the exchange. Therefore, such patients or patients with comorbidities must be monitored while the tube is being exchanged and supplemental oxygen must be readily available.

In our case, the imperfect positioning of the tracheostomy tube with subsequent forceful ventilation via an Ambu® bag (Ambu A/S, Copenhagen, Denmark) most likely led to extraluminal air, resulting in subcutaneous emphysema, pneumothorax, pneumomediastinum, and pneumoperitoneum. An upright chest X-ray can easily diagnose the above findings, but a CT can more accurately detect pneumomediastinum and pneumoperitoneum by delineating the spread of air between the chest, abdomen, and pelvis.

Pneumomediastinum without pneumothorax is usually treated conservatively. Pneumoperitoneum frequently requires emergency surgery. If there is no clinical evidence of organ perforation, it can be managed conservatively, which accounts for 10-15% cases of pneumoperitoneum [4]. In our patient, ruling out surgical abdomen was not possible without linking the tracheostomy tube exchange with tension pneumothorax and pneumoperitoneum.

## Conclusions

Simple and straight forward procedures, like an exchange of a tracheotomy tube after a preformed tract between skin and trachea, can lead to fatal complications if not handled properly. The diagnosis can be challenging, especially in nursing home patients with multiple comorbidities. Understanding the anatomic spaces and how they communicate with each other helps with the diagnosis. The outcome was favorable along with focused management and supportive treatment.
